# Comparison of genome replication fidelity between SARS-CoV-2 and influenza A virus in cell culture

**DOI:** 10.1038/s41598-023-40463-4

**Published:** 2023-08-11

**Authors:** Yoshiko Kawasaki, Haruka Abe, Jiro Yasuda

**Affiliations:** 1 Department of Emerging Infectious Diseases, National Research Center for the Control and Prevention of Infectious Diseases (CCPID), Nagasaki University, 1-12-4 Sakamoto, Nagasaki, Nagasaki 852-8523 Japan; 2 Department of Emerging Infectious Diseases, Institute of Tropical Medicine (NEKKEN), Nagasaki University, 1-12-4 Sakamoto, Nagasaki, Nagasaki 852-8523 Japan; 3 Graduate School of Biomedical Science, Nagasaki University, 1-12-4 Sakamoto, Nagasaki, Nagasaki 852-8523 Japan

**Keywords:** Virology, Influenza virus, SARS-CoV-2

## Abstract

Since the emergence of COVID-19, several SARS-CoV-2 (severe acute respiratory syndrome coronavirus 2) variants have emerged and spread widely. These variants are produced through replication errors of the viral genome by viral RNA-dependent RNA polymerase (RdRp). Seasonal epidemics of influenza are also known to occur because of new variants of influenza A virus (IAV), which are generated by the introduction of mutations by viral RdRp with low fidelity. Variants with different antigenicities appear because of mutations in envelope glycoproteins. In this study, we calculated and compared the mutation rates in genome replication of IAV and SARS-CoV-2. Average mutation rates per passage were 9.01 × 10^–5^ and 3.76 × 10^–6^ substitutions/site for IAV and SARS-CoV-2, respectively. The mutation rate of SARS-CoV-2 was 23.9-fold lower than that of IAV because of the proofreading activity of the SARS-CoV-2 RdRp complex. Our data could be useful in establishing effective countermeasures against COVID-19.

## Introduction

In December 2019, the coronavirus disease 2019 (COVID-19), caused by severe acute respiratory syndrome coronavirus 2 (SARS-CoV-2), emerged in Wuhan, China, and then spread worldwide. Many variants of SARS-CoV-2 have appeared since its first isolation, causing a continuous pandemic in the world.

Influenza A virus (IAV) infection causes annual seasonal epidemics and pandemics. Seasonal influenza is caused by new variants with antigenicities different from those of the previous epidemic strain, which emerged by the accumulation of mutations introduced through replication errors of the viral genome by viral RNA-dependent RNA polymerase (RdRp), so called “antigenic drift”.

Both SARS-CoV-2 and IAV are enveloped RNA viruses that cause respiratory disease. SARS-CoV-2 is a positive-stranded RNA virus, with a genome of approximately 29.8 kilobases, which is annotated to encode 14 ORFs (Open Reading Frames) and 27 proteins^1^, whereas IAV is a negative-stranded RNA virus, with eight segmented genomes of approximately 13.6 kilobases, which encode at least 10 viral proteins^2^. The RdRp of most RNA viruses, including IAV, has few proofreading abilities, and its fidelity in genome replication is low. Therefore, the mutation rates of RNA viruses are generally much higher than those of DNA viruses.

However, RdRp complexes of coronaviruses, including SARS-CoV, are reported to have proofreading activity owing to 3′-to-5′ exoribonuclease activity of the viral protein, nsp14, in their genome replication^3,4^. SARS-CoV-2 also has nsp14 with proofreading activity^5^.

Influenza A virus hemagglutinin (HA), neuraminidase (NA), and SARS-CoV-2 S proteins are viral surface glycoproteins and the main determinants of viral antigenicity, which are recognized by the host immune system. Influenza and COVID-19 epidemics and pandemics are caused by variants with antigenicities different from those of previous epidemic or pandemic strains through the introduction of mutations into these viral surface glycoproteins. Therefore, investigating the mutation rates of the HA and NA genes of IAV and S gene of SARS-CoV-2 is very important for understanding the appearance of new epidemic variants and establishing appropriate countermeasures against these viral diseases.

The extended duration of the COVID-19 pandemic and increasing number of SARS-CoV-2 infections are likely to give rise to variants of the virus with new characteristics in terms of pathogenicity, antigenicity, and tropism, making this infectious disease difficult to control.

Here, we determined the in vitro mutation rates of IAV and SARS-CoV-2 in the absence of any immune pressure and analysed the characteristics of the mutations introduced into the IAV HA/NA and SARS-CoV-2 S genes.

## Results

### Calu-3 cells are susceptible to both SARS-CoV-2 and influenza A virus infection

**Figure 1:**
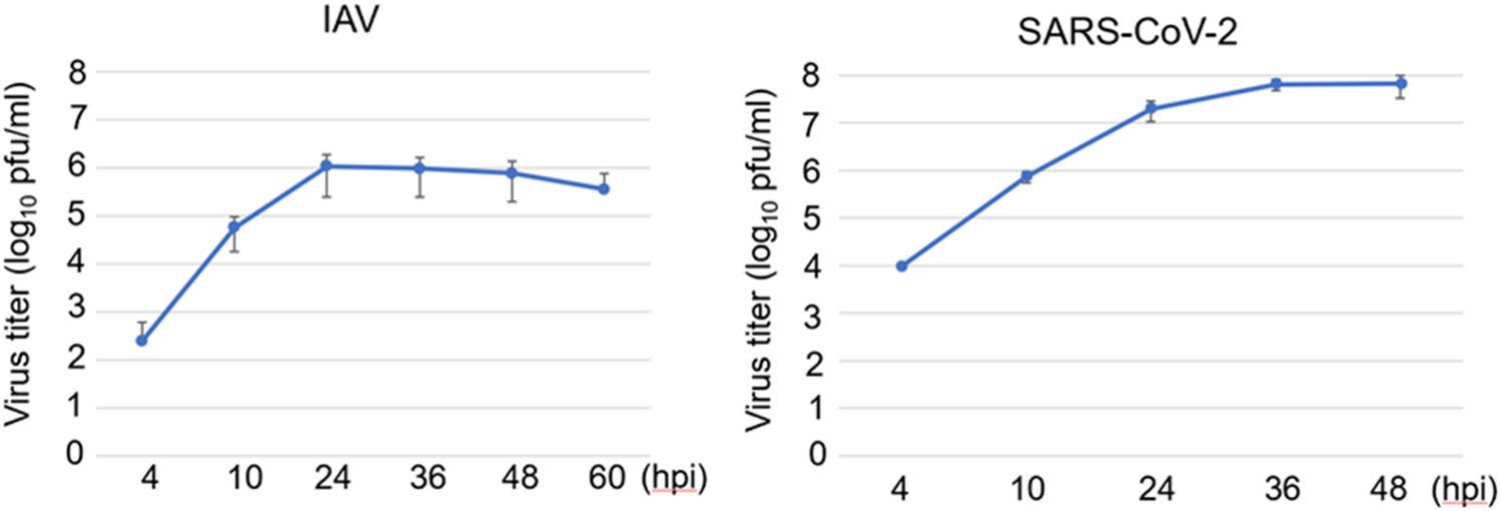
Similar growth kinetics between IAV and SARS-CoV-2 in Calu-3 cells. Calu-3 cells were infected with IAV or SARS-CoV-2 at a multiplicity of infection (MOI) of 1. Culture supernatants were collected at the indicated times, and virus titres were measured by plaque assay. The bar plot represents the mean ± SD. *hpi* hours post infection.

First, we examined the growth kinetics of SARS-CoV-2 and IAV. Calu-3 cells, an adenocarcinoma cell line derived from human lung epithelial cells and susceptible to both viruses^6–9^, were used for this analysis, as the tested viruses are RNA viruses that cause acute respiratory diseases. Calu-3 cells were inoculated with IAV or SARS-CoV-2 at a multiplicity of infection (MOI) of 1. The titres of the progeny viruses were measured using a plaque assay. Both viruses showed similar growth kinetics and reached a plateau at 24–36 h post infection (hpi) (Fig. 1). The virus production yields of IAV at 24 hpi and SARS-CoV-2 at 36 hpi were 1.1 × 10^6^ and 6.5 × 10^7^ pfu/mL, respectively.

### Mutation rate of SARS-CoV-2 in vitro is much lower than that of IAV

To calculate the mutation rates in the genome replication of IAV and SARS-CoV-2, each virus was serially passaged every 48 h in Calu-3 cells. After 15 serial passages, culture supernatants were collected and clarified by centrifugation. Viral RNA was extracted from the supernatant after centrifugation and used for genetic analysis. For each virus, three independent lines of passages (P15-A, B, and C) were used.

**Figure 2:**
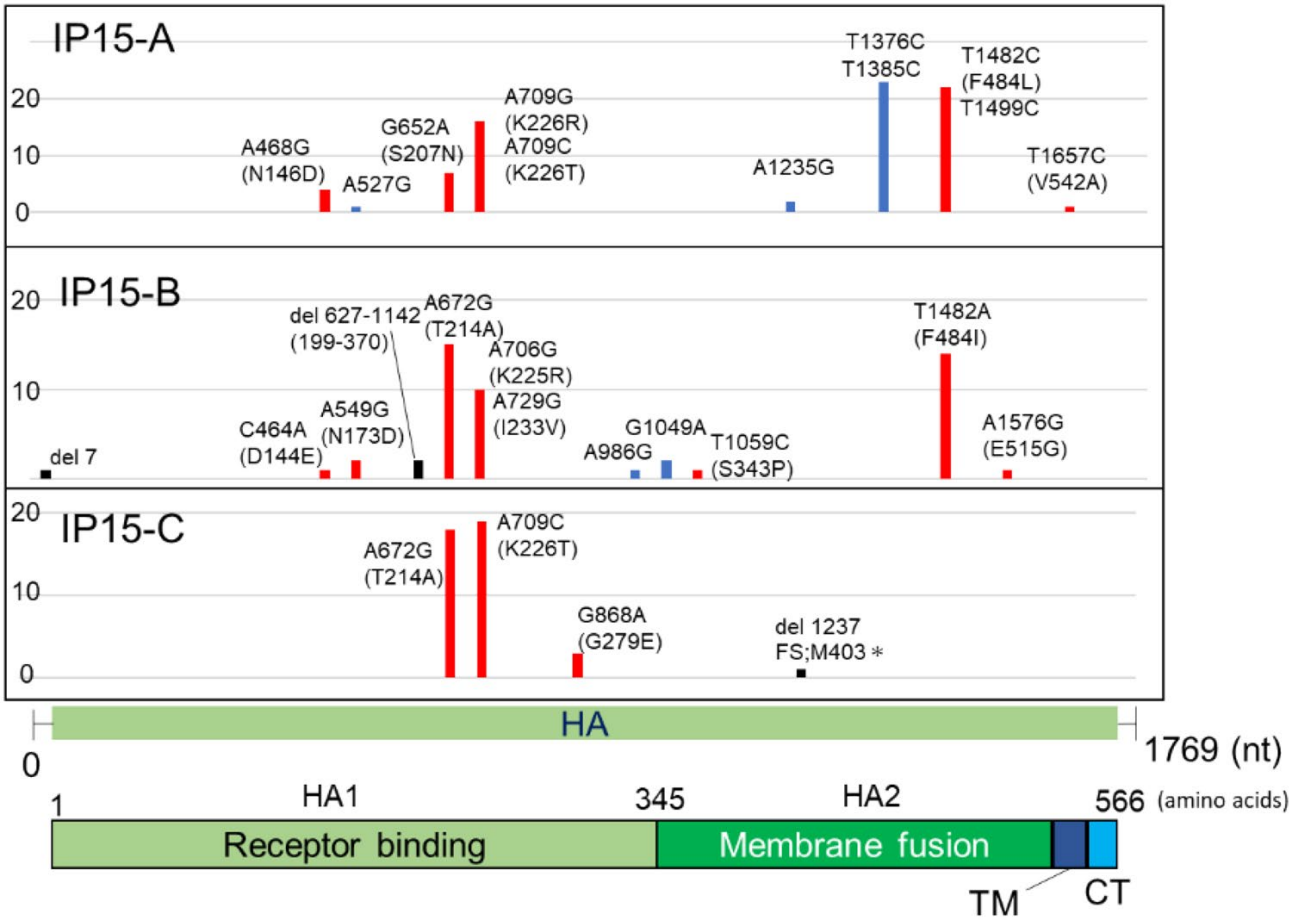
Mutations detected in IAV HA gene after 15 serial passages in Calu-3 cells. The graph shows the position and frequency of mutations in IAV HA gene. The vertical axis shows the number of clones in which mutations were observed among the 20 clones, and the horizontal axis indicates the position at which the mutation occurred. HA gene structure and protein domains are shown below the graph. Colours indicate the type of mutations: blue, synonymous mutations; red, non-synonymous mutations, black: deletions. *TM* transmembrane, *CT* cytoplasmic tail region.

**Figure 3:**
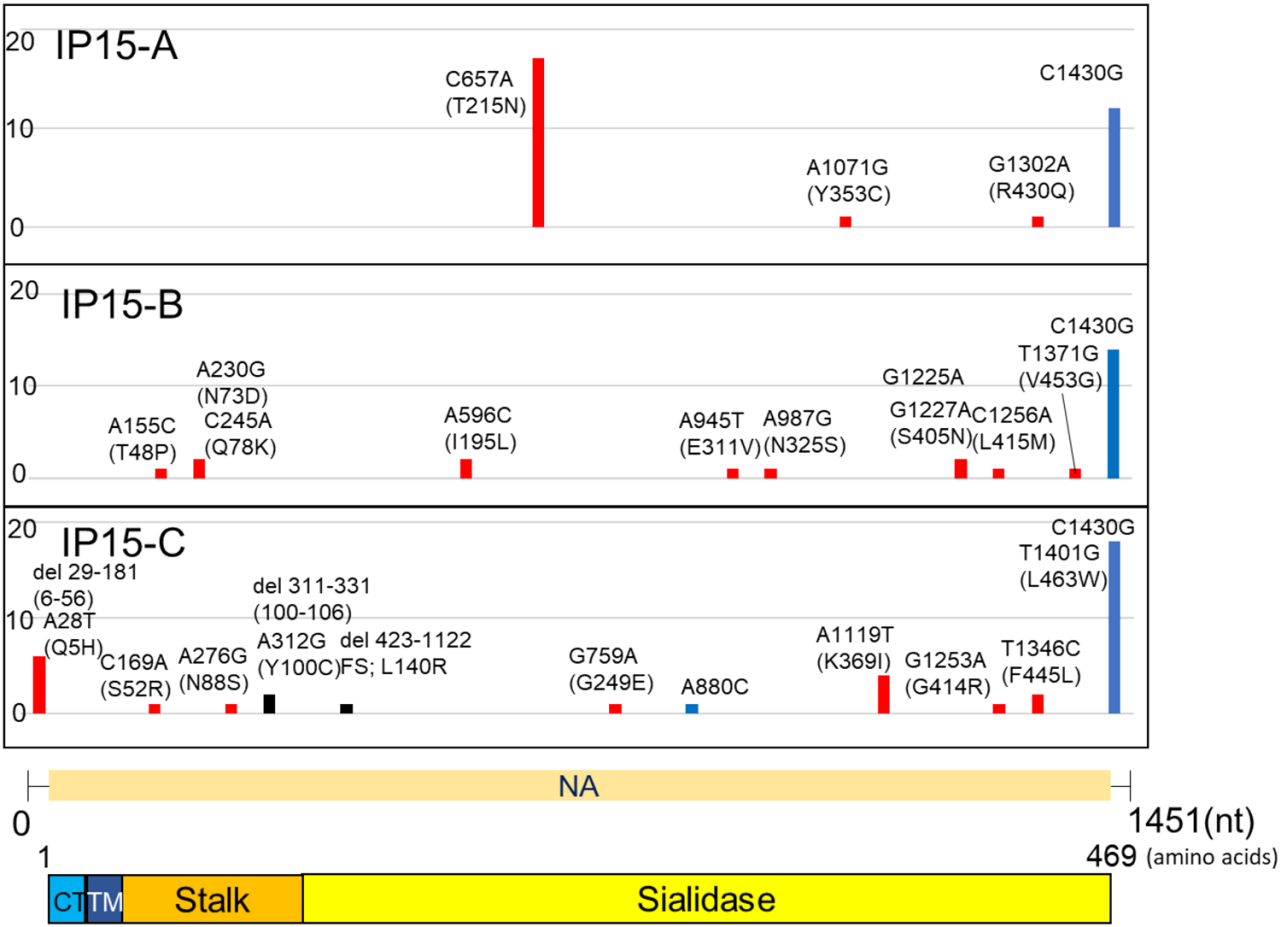
Mutations detected in IAV NA gene after 15 serial passages in Calu-3 cells. The graph shows the position and frequency of mutations in NA gene. The vertical axis shows the number of clones in which mutations were observed among the 20 clones, and the horizontal axis indicates the position at which the mutation occurred. NA gene structure and protein domains are shown below the graph. Colours indicate the type of mutations: blue, synonymous mutations; red, non-synonymous mutations, black: deletions. *TM* transmembrane, *CT* cytoplasmic tail region.

**Figure 4:**
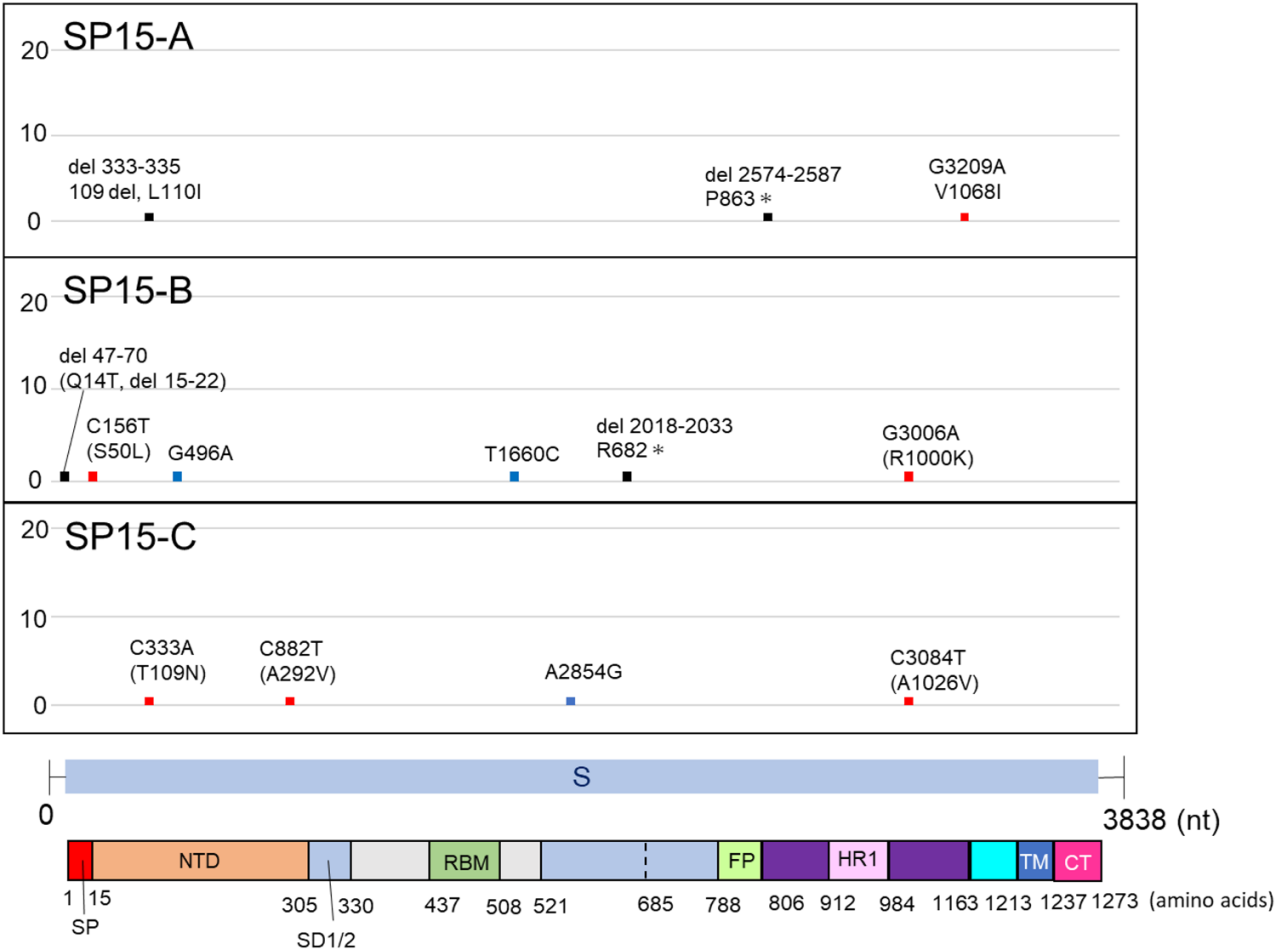
Mutations detected in SARS-CoV-2 S gene after 15 serial passages in Calu-3 cells. The graph shows the position and frequency of mutations in SARS-CoV-2 S gene. The graph shows the position and frequency of mutations in S gene. The vertical axis shows the number of clones in which mutations were observed among the 20 clones, and the horizontal axis indicates the position at which the mutation occurred. S gene structure and protein domains are shown below the graph. Colours indicate the type of mutations: blue, synonymous mutations; red, non-synonymous mutations, black: deletions. *SP* signal peptide, *NTD* N-terminal domain, *RBD* receptor-binding domain, *RBM* receptor-binding motif, *SD1/2* subdomain 1 and 2, *FP* fusion peptide, *HR1* heptad repeat regions 1.

The HA [1769 nucleotides (nt)] and NA (1451 nt) genes for IAV and S gene (3838 nt) for SARS-CoV-2, which encode viral surface glycoproteins, were selected for gene sequence determination. Based on the RNA samples, after 15 passages of SARS-CoV-2 (SP15-A, -B and -C) and IAV (IP15-A, -B and -C), the HA and NA genes for IAV and S genes for SARS-CoV-2 were amplified by Reverse Transcription Polymerase Chain Reaction (RT-PCR) and then cloned into the plasmids. The nucleotide sequences of 20 clones were determined for each RNA sample. The positions of the mutations introduced until passage 15 and the number of clones with the mutation are shown in Figs. 2, 3, and 4.

Regarding IAV, mutations were observed at 24 sites in HA and 29 sites in NA in the three passage lines after 15 passages (IP15-A, -B, and -C). The mutations were highly frequent between 650 and 730 nt in HA (Fig. 2). The mutation at 1482 bp was also dominant in IP15-A and B. In contrast, there were no common mutations among the three passage lines in the coding region of NA (Fig. 3). The only mutation site found in all three passage lines was the C to G mutation at nucleotide position 1430 within the untranslated region. The number of mutation sites in the SARS-CoV-2 S gene was 13, which was much lower than that in the IAV HA and NA genes (Fig. 4). No common mutations were observed among the three passage lines of SARS-CoV-2.

**Figure 5:**
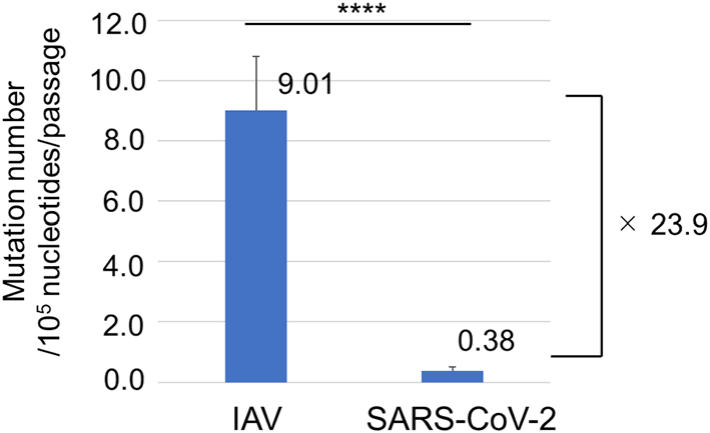
Number of mutations including deletions and insertions per 10^5^ nucleotides/passage. The average number of mutations after 15 serial passages of IAV and SARS-CoV-2 are shown. The vertical axis represents the number of mutations per 10^5^ nucleotides per passage. IAV nucleotides include HA and NA gene sequences, and SARS-CoV-2 refers to S gene sequences. The bar plot represents the mean ± SD. ****p < 0.0001 (Mann–Whitney-U test).

The 20 clones of IAV passage lines (IP15-A, -B, and -C) contained 76, 50, and 41 mutations in HA, and 31, 25, and 38 mutations in NA, respectively, whereas those of SARS-CoV-2 (SP15-A, -B, and -C) showed 3, 6, and 4 mutations in the S gene, respectively. The mutation rates were calculated based on this information and the data are summarized in Table 1. The average of mutation rates (mutations per nucleotide) of three passage lines after 15 passages were 1.35 × 10^–3^ (± 4.07 × 10^–4^) substitutions/site for IAV and 5.65 × 10^–5^ (± 1.63 × 10^–5^) for SARS-CoV-2. The average mutation rates per passage were 9.01 × 10^–5^ (± 2.71 × 10^–5^) substitutions/site/passage for IAV and 3.76 × 10^–6^ (± 1.09 × 10^–6^) for SARS-CoV-2, indicating that the mutation rate of SARS-CoV-2 was 23.9-fold lower than that of IAV (Fig. 5).

**Table 1:** Mutation rate of each viral gene after 15 serial passages and per passage. After 15 serial passages, the number of substitutions, including indels, was counted in each of the 20 clones of the three different passage lines of IAV (IP15) and SARS-CoV-2 (SP15). The average of each passage line was calculated and the substitution numbers were compared. Values represent mean ± SD. *p* < 0.01 (Mann–Whitney-U test).

Clone	Gene	After 15 passages			Per passage	
		After P15		Average	Average	*P* value
IP15-A	HA	2.15 × 10^–3^	1.57 × 10^–3^ (± 4.20 × 10^–4^)	1.35 × 10^–3^ (± 4.07 × 10^–4^)	9.01 × 10^–5^ (± 2.71 × 10^–5^)	0.0021
IP15-B	HA	1.41 × 10^–3^				
IP15-C	HA	1.16 × 10^–3^				
IP15-A	NA	1.07 × 10^–3^	1.08 × 10^–3^ (± 1.83 × 10^–4^)			
IP15-B	NA	8.61 × 10^–4^				
IP15-C	NA	1.31 × 10^–3^				
SP15-A	S	3.91 × 10^–5^		5.65 × 10^–5^ (± 1.63 × 10^–5^)	3.76 × 10^–6^ (± 1.09 × 10^–6^)	
SP15-B	S	7.82 × 10^–5^				
SP15-C	S	5.21 × 10^–5^

### Frequencies of synonymous and non-synonymous mutations differ between IAV and SARS-CoV-2

Next, we calculated the frequencies (mutation rates) of synonymous and non-synonymous mutations in the coding regions and insertions/deletions (indels) in each viral gene. The results are presented in Table 2. The average frequencies of non-synonymous and synonymous mutations were 1.21 × 10^–3^ (± 7.78 × 10^–5^) and 4.02 × 10^–4^ (± 5.97 × 10^–4^) substitutions/site for IAV HA, 5.32 × 10^–4^ (± 1.63 × 10^–4^) and 5.08 × 10^–4^ (± 1.08 × 10^–4^) for IAV NA, and 1.31 × 10^–5^ (± 0.00) and 1.31 × 10^–5^ (± 1.31 × 10^–5^) for SARS-CoV-2 S, respectively. The ratios of non-synonymous to synonymous mutations (dN/dS) in HA, NA, and S genes were 3.0, 1.0, and 1.0, respectively, suggesting a strong positive selection for HA.

**Table 2:** Mutation rate of each viral gene that occurred as non-synonymous mutations, synonymous mutations, and insertions/deletions. The number of synonymous and non-synonymous substitutions was counted in the coding region: HA: 33–1733 nt; NA: 14–1423 nt; S: 17–3822 nt. Values represent mean ± SD. *p* < 0.01 (Mann–Whitney-U test).

Clone	Gene	Non-synonymous				Synonymous				Insertions/deletions			
		After P15		Average	P value	After P15		Average	P value	After P15		Average	P value
IP15-A	HA	1.15 × 10^–3^	1.21 × 10^–3^ (± 7.78 × 10^–5^)	9.00 × 10^–4^ (± 3.21 × 10^–5^)	0.0076	1.09 × 10^–3^	4.02 × 10^–4^ (± 5.97 × 10^–4^)	4.50 × 10^–4^ (± 2.79 × 10^–4^)	0.0987	0	2.94 × 10^–5^ (± 2.94 × 10^–5^)	4.82 × 10^–5^ (± 4.25 × 10^–5^)	0.4485
IP15-B	HA	1.29 × 10^–3^				1.18 × 10^–4^				5.65 × 10^–5^			
IP15-C	HA	1.18 × 10^–3^				0				2.83 × 10^–5^			
IP15-A	NA	6.74 × 10^–4^	5.32 × 10^–4^ (± 1.63 × 10^–4^)			3.90 × 10^–4^	5.08 × 10^–4^ (± 1.08 × 10^–4^)			3.45 × 10^–5^	7.09 × 10^–5^ (± 9.38 × 10^–5^)		
IP15-B	NA	3.55 × 10^–4^				5.32 × 10^–5^				0			
IP15-C	NA	5.67 × 10^–4^				6.03 × 10^–4^				1.72 × 10^–4^			
SP15-A	S	1.31 × 10^–5^		1.31 × 10^–5^ (± 0)		0		1.31 × 10^–5^ (± 1.31 × 10^–5^)		2.61 × 10^–5^		1.75 × 10^–5^ (± 1.52 × 10^–5^)	
SP15-B	S	1.31 × 10^–5^				2.63 × 10^–5^				2.61 × 10^–5^			
SP15-C	S	1.31 × 10^–5^				1.31 × 10^–5^				0			

In IAV HA, non-synonymous mutations were concentrated in the receptor-binding domain (RBD) (Fig. 2). The non-synonymous mutations A672G (amino acid position: T214A) and A709G/A709C (amino acid position: K226R/K226T) were observed in many clones, suggesting that these amino acid substitutions are involved in the acquisition of growth advantage in Calu-3 cells. In the SARS-CoV-2 S gene, synonymous and non-synonymous mutations were scattered in the gene (Fig. 4).

### Mutations are mostly transitions in the SARS-CoV-2 S gene

**Figure 6:**
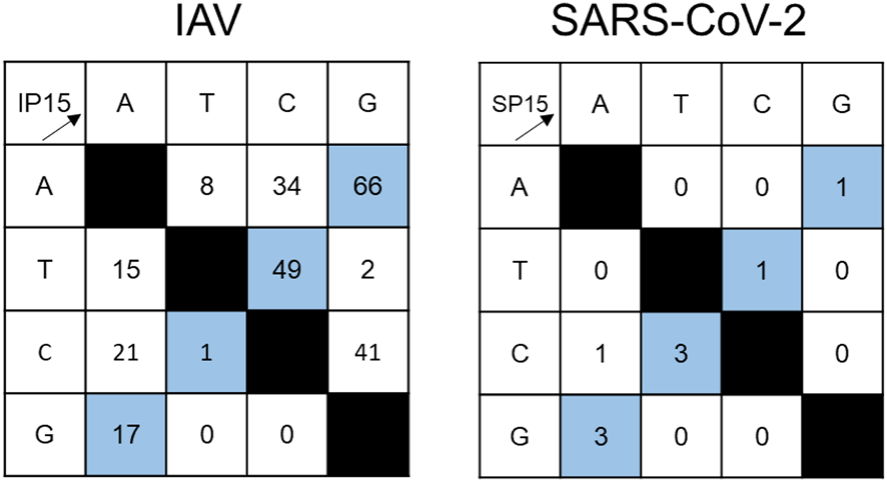
Nucleotide substitution in IAV and SARS-CoV-2 after 15 serial passages. Nucleotide substitutions were counted in IAV and SARS-CoV-2 after 15 serial passages. IP15 and SP15 indicate 15 serial passage lines of IAV and SARS-CoV-2, respectively. Colours indicate the type of substitutions: blue, transition; white, transversion.

We further analysed the mutations introduced into the HA, NA, and S genes for transition and transversion mutations. The total numbers of transitions and transversions were 133 and 121 in the IAV HA and NA genes and eight and one in the SARS-CoV-2 S gene, respectively (Fig. 6). Thus, the frequencies of transition and transversion were similar in IAV, whereas most mutations in SARS-CoV-2 were transitions.

### Indels frequencies are similar between IAV and SARS-CoV-2

The frequencies of indels in IAV HA and NA genes were 2.94 × 10^–5^ (± 2.94 × 10^–5^) and 7.09 × 10^–5^ (± 9.38 × 10^–5^) substitutions/site, respectively, and the average frequency for both genes was 4.82 × 10^–5^ (± 4.25 × 10^–5^) substitutions/site (Table 2). In contrast, that of the SARS-CoV-2 S gene was 1.75 × 10^–5^ (± 1.52 × 10^–5^) substitutions/site. There was no significant difference in the frequency of indels between IAV and SARS-CoV-2.

## Discussion

In this study, we showed that the mutation rate of IAV was 9.01 × 10^–5^ substitutions/site/passage (that is, 4.51 × 10^–5^ substitutions/site/lifecycle with an IAV lifecycle of 24 h), whereas a previous study reported that those in the NS genes of two IAV strains, A/Aichi/1/87 (H1N1) and A/Aichi/12/92 (H3N2), in MDCK (Madin-Darby canine kidney) cells were 1.8 × 10^–5^ and 0.8 × 10^–5^ substitutions/site/lifecycle^10^. The differences in the mutation rates may be due to differences in virus strains, viral genes, and cells, which were used for analyses. In the present study, we used the pandemic IAV strain A/Hyogo/YS/2011 (H1N1pdm09) and Calu-3 cells and analysed HA and NA genes.

Our results revealed that the fidelity of SARS-CoV-2 genome replication was 23.9-fold higher than that of IAV. This higher fidelity of the SARS-CoV-2 RdRp complex is thought to be mainly due to the proofreading activity of the 3′-to-5′ exoribonuclease activity of the viral protein, nsp14^5^. In contrast, there was no significant difference in the frequencies of indels between IAV and SARS-CoV-2, suggesting that SARS-CoV-2 does not have a special mechanism to prevent insertion and deletion in its genome replication and the process works as well as that in IAV.

In the human population, the evolutionary rates of IAV HA and NA genes were reported to be 4.84 × 10^–3^ and 3.87 × 10^–3^ substitutions/site/year, respectively^11^, whereas that of the SARS-CoV-2 S gene was previously reported to be 0.81–1.08 × 10^–3^ substitutions/site/year^12,13^, indicating an approximately 4.93-fold higher evolutionary rate of IAV than that of SARS-CoV-2. The annual number of cases of COVID-19 has been reported to be 85 million in 2020, 200 million in 2021, and 315 million for nine months in 2022^14^, whereas that of seasonal influenza before the COVID-19 pandemic has been estimated to be 350 million to one billion per year^15^. Considering that the number of cases of seasonal influenza is greater than that of COVID-19 and that the fidelity of SARS-CoV-2 genome replication is much higher than that of IAV, the difference in the evolutionary rates between SARS-CoV-2 and IAV seems to be much less than expected according to the results of the present study. A longer infection period and higher replication numbers of SARS-CoV-2 in infected individuals may result in a higher evolutionary rate in humans than in vitro.

The ratio of non-synonymous/synonymous mutations (dN/dS) was 1.0 in IAV NA and SARS-CoV-2 S genes, whereas that of IAV HA gene was 3.0. This strongly suggests the positive selection for HA during serial passaging in Calu-3 cells. Most non-synonymous mutations in HA were observed in the RBDs (Fig. 2). Non-synonymous mutations A672G (amino acid position: T214A) and A709G/A709C (amino acid position: K226R/K226T) were observed in many clones. Among RBDs, the amino acid residues 200–241 have been reported to affect the binding affinity and specificity to the host cellular receptors^16–19^. Therefore, amino acid substitutions at positions 214 and 226 are likely to be involved in the acquisition of the growth advantage of IAV in Calu-3 cells.

This positive selection of the HA gene may affect the calculation of the mutation rate of the HA gene. Therefore, we further compared the frequencies of synonymous mutations, which have no effect on protein function. The frequencies of synonymous mutations in the HA and NA genes after 15 passages were 4.02 × 10^–4^ and 5.08 × 10^–4^ substitutions/site, respectively, and they were not significantly different (Table 2). In contrast, that of the SARS-CoV-2 S gene was 1.31 × 10^–5^ substitutions/site and was 30.7- or 38.8-fold lower than that of HA or NA, respectively. This indicates that the mutation rate of the S gene was significantly lower than that of the HA and NA genes, even with the bias of positive selection for the HA gene being considered.

We observed that most mutations in the S gene of SARS-CoV-2 after 15 passages were transitions. This may be a feature of the RdRp of SARS-CoV-2, as it is consistent with a previous report that the major mutational type found in the complete genome of SARS-CoV-2 strains identified worldwide was transitions^20^.

Taken together, we revealed that the in vitro mutation rate of SARS-CoV-2 is much lower than that of IAV because of the proof-reading activity of the SARS-CoV-2 RdRp complex. This suggests that the reduction in the number of infected cases prevents the appearance of new variants with different antigenicities, increased infectivity, or higher pathogenicity, and would be important to terminate the COVID-19 pandemic.

## Methods

### Cells

VeroE6 cells stably expressing human TMPRSS2 (Vero/TMPRSS2 cells) were kindly provided by Dr. Shutoku Matsuyama (National Institute of Infectious Diseases, Japan)^21^. The cells were obtained from the Japanese Collection of Research Bioresources Cell Bank in Japan (JCRB no. JCRB1819). Vero/TMPRSS2 and Calu-3 cells were maintained in Dulbecco’s modified Eagle’s medium (DMEM; Fujifilm Wako, Osaka, Japan) supplemented with 10% fetal bovine serum (FBS) and 1% penicillin/streptomycin solution (Gibco, Thermo Fisher Scientific, Waltham, MA, USA) at 37 °C with 5% CO_2_. Madin-Darby canine kidney (MDCK) cells were maintained in modified Eagle’s medium (MEM; Gibco, Thermo Fisher Scientific) supplemented with 5% FBS and 1% penicillin/streptomycin solution at 37 °C with 5% CO_2_.

### Viruses and virus titration

Influenza A virus (IAV) A/Hyogo/YS/2011 (H1N1pdm09) and SARS-CoV-2 JPN/NGS/SC-1/2020 (GISAID Accession ID: EPI_ISL_481254) were used. To isolate a single clone of each virus strain, a single plaque isolation was performed three times in MDCK cells for IAV and twice in Vero/TMPRSS2 cells for SARS-CoV-2.

IAV or SARS-CoV-2 clones isolated from a single plaque were propagated in MDCK cells in MEM containing 1 × Vitamin (Gibco, Thermo Fisher Scientific), 0.1% BSA (Gibco, Thermo Fisher Scientific), MEM non-essential amino acids (NEAA; Gibco, Thermo Fisher Scientific), and 0.00075% Trypsin (Gibco, Thermo Fisher Scientific), or Vero/TMPRSS2 cells in DMEM containing 1% FBS^22^.

Plaque assays for IAV were performed as previously described^23,24^. For SARS-CoV-2, a plaque assay was performed with monolayer cultures of Vero/TMPRSS2 cells on a 12-well plate. After virus adsorption, cells were over-layered with 1 × MEM containing 0.7% agarose, 1.5% FBS, and 1% penicillin/streptomycin solution and then incubated at 37 °C with 5% CO_2_ for 2 days. The cells were fixed with ethanol: acetic acid (5:1) for 2 h at 24 °C and then stained with an amido-black solution. For both viruses, a single plaque isolation was performed before fixation.

All experiments using IAV or SARS-CoV-2 were performed in a biosafety level (BSL)-2 or BSL-3 laboratory at Nagasaki University.

### Analyses of growth kinetics of IAV and SARS-CoV-2 in Calu-3 cells

Calu-3 cells (5 × 10^5^ cells/well) were seeded in 24-well plates. After 24 h, cells were infected with IAV or SARS-CoV-2 at a multiplicity of infection (MOI) of 1. Culture supernatants were collected at the indicated times after infection, and the titres of progeny viruses were measured by plaque assay.

### Serial blind passages of the viruses

Calu-3 cells (1.6 × 10^6^ cells/well) were seeded in 6-well plates. After 24 h, the cells were infected with IAV or SARS-CoV-2 at an MOI of 0.1 and then incubated for 48 h. After incubation, the culture supernatants were collected and centrifuged (10,000 rpm at 4 °C for 5 min) to sediment cell debris. The supernatants were diluted 10 times with DMEM and then inoculated into freshly prepared Calu-3 cells in 6-well plates. This process was repeated 15 times. Serial viral passages were independently performed in three lines for each virus.

### Viral RNA extraction and RT-PCR

Viral RNA was extracted from the supernatants after each passage using the QIAmp Viral RNA Mini Kit (Qiagen, Hilden, Germany), according to the manufacturer’s protocol. The IAV HA and NA genes and SARS-CoV-2 S gene were amplified by RT-PCR using the PrimeScript II High Fidelity One Step RT-PCR Kit (Takara Bio, Shiga, Japan). Primers used to amplify each viral gene are listed in Supplementary Table S1.

### Sequence determination

Each viral gene amplified by RT-PCR was ligated to the pCR-Blunt II-TOPO vector using the Zero Blunt TOPO PCR Cloning Kit (Invitrogen, Thermo Fisher Scientific, Carlsbad, CA) and then transformed into *Escherichia coli* DH5α competent cells (Takara Bio). The transformants were plated on LB plates containing ampicillin. After incubation for 18 h, 20 colonies were picked and the nucleotide sequences of the HA, NA, or S genes in each clone were determined using the primers listed in Supplementary Table 1.

### Quantification and statistical analysis

All graphs are plotted as mean ± standard deviation. The data were analysed using Graph Pad Prism version 9.4.1 (Graph Pad, San Diego, CA). Statistical significance was determined using the Student’s *t*-test, as indicated in the figure legends. Statistical significance was set at *p* < 0.05. In all figures, ∗ denotes *p* < 0.05, ∗∗*p* < 0.01, ∗∗∗*p* < 0.001, and ∗∗∗∗*p* < 0.0001.

### Supplementary Information

Supplementary Table S1.

## Data Availability

Data described in this manuscript will be made available upon request and approval by the principal investigator, Jiro Yasuda (j-yasuda@nagasaki-u.ac.jp).
