# Supplementary material for: Comparison of genome replication fidelity between SARS-CoV-2 and influenza A virus in cell culture
Source: Sci Rep. 2023 Aug 11;13:13105. doi: 10.1038/s41598-023-40463-4 (PMC10421855; doi:10.1038/s41598-023-40463-4)
Supplement: Supplementary file 1 — Supplementary Table S1. [file 41598_2023_40463_MOESM1_ESM.pdf]

Supplementary Table 1. Primer sequences used in this study

Target	Oligo name	Oligo Sequence
For RT-PCR		
IAV(HA)	F1 Primer(H1N1-HA)	AGCAAAAGCAGGGGAAAACAAAAGC
	R1 Primer(H1N1-HA)	CAAGGGTGTTTTTCTCATGC
IAV(NA)	F1 Primer(H1N1-NA)	GCAGGAGTTTAAAATGAATCC
	R1 Primer(H1N1-NA)	AGTAGAAACAAGGAGTTTTTTCAACG
SARS-CoV2(S)	F1 Primer(SARS-CoV-2 S gene)	CAAACCACGCGAACAAATAG
	R1 Primer(SARS-CoV-2 S gene)	AACGCCAACAATAAGCCATC
For PCR		
IAV(HA)	F1' Primer(H1N1-HA)	GGAACGTGTTACCCAGGAGA
	F2' Primer(H1N1-HA)	CACCCAAGGGTGCTATAAACA
	F3' Primer(H1N1-HA)	ATGGTTTCCTGGACATTTGG
	R1' Primer(H1N1-HA)	GCTGCCGTTACACCTTTGTT
IAV(NA)	F1' Primer(H1N1-NA)	CCATTGGTTCGGTCTGTATG
	F2' Primer(H1N1-NA)	TGAGTCAGTCGCTTGGTCAG
	F3' Primer(H1N1-NA)	AGACAATCCACGCCCTAATG
	R1' Primer(H1N1-NA)	GGGGAGCATGATATGAATGG
SARS-CoV2(S)	F1' Primer(SARS-CoV-2 S gene)	CAAACCACGCGAACAAATAG
	F2' Primer(SARS-CoV-2 S gene)	TCGAAGACCCAGTCCCTACTT
	F3' Primer(SARS-CoV-2 S gene)	TGCCCTTTTGGTGAAGTTTT
	F4' Primer(SARS-CoV-2 S gene)	CTTCCCTCAGTCAGCACCTC
	F5' Primer(SARS-CoV-2 S gene)	GTGGTCAACCAAAATGCACA
	F6' Primer(SARS-CoV-2 S gene)	GCACAGAAGTCCCTGTTGCT
	F7' Primer(SARS-CoV-2 S gene)	CAGGGTTTTTCGGCTTTAGA
	F8' Primer(SARS-CoV-2 S gene)	CCGTGCTTTAACTGGAATAGC
	F9' Primer(SARS-CoV-2 S gene)	ATTTAGGTGACACTATAG
	F10' Primer(SARS-CoV-2 S gene)	ACCCACTAATGGTGTTGGTTAC
	R1' Primer(SARS-CoV-2 S gene)	GAGAGAGGGTCAAGTGCACAG
	R2' Primer(SARS-CoV-2 S gene)	AACGCCAACAATAAGCCATC
	R3' Primer(SARS-CoV-2 S gene)	CCAACTTTTGTTGTTTTTGTGG
	F Primer(M13)	GTAAAACGACGGCCAG
	R Primer(M13)	CAGGAAACAGCTATGAC
